# Role of hydrogen sulphide in physiological and pathological angiogenesis

**DOI:** 10.1111/cpr.13374

**Published:** 2022-12-07

**Authors:** Yan‐Xia Zhang, Mi‐Rong Jing, Chun‐Bo Cai, Shuai‐Gang Zhu, Chao‐Jing Zhang, Qi‐Meng Wang, Yuan‐Kun Zhai, Xin‐Ying Ji, Dong‐Dong Wu

**Affiliations:** ^1^ Henan International Joint Laboratory for Nuclear Protein Regulation, School of Basic Medical Sciences Henan University Kaifeng Henan China; ^2^ Kaifeng Municipal Key Laboratory of Cell Signal Transduction, Henan Provincial Engineering Centre for Tumor Molecular Medicine Henan University Kaifeng Henan China; ^3^ School of Stomatology Henan University Kaifeng Henan China; ^4^ Kaifeng Key Laboratory of Infection and Biological Safety, School of Basic Medical Sciences Henan University Kaifeng Henan China

## Abstract

The role of hydrogen sulphide (H_2_S) in angiogenesis has been widely demonstrated. Vascular endothelial growth factor (VEGF) plays an important role in H_2_S‐induced angiogenesis. H_2_S promotes angiogenesis by upregulating VEGF via pro‐angiogenic signal transduction. The involved signalling pathways include the mitogen‐activated protein kinase pathway, phosphoinositide‐3 kinase pathway, nitric oxide (NO) synthase/NO pathway, signal transducer and activator of transcription 3 (STAT3) pathway, and adenosine triphosphate (ATP)‐sensitive potassium (K_ATP_) channels. H_2_S has been shown to contribute to tumour angiogenesis, diabetic wound healing, angiogenesis in cardiac and cerebral ischaemic tissues, and physiological angiogenesis during the menstrual cycle and pregnancy. Furthermore, H_2_S can exert an anti‐angiogenic effect by inactivating Wnt/β‐catenin signalling or blocking the STAT3 pathway in tumours. Therefore, H_2_S plays a double‐edged sword role in the process of angiogenesis. The regulation of H_2_S production is a promising therapeutic approach for angiogenesis‐associated diseases. Novel H_2_S donors and/or inhibitors can be developed in the treatment of angiogenesis‐dependent diseases.

## Graphical abstract

Hydrogen sulphide and nitric oxide interact and depend on each other to jointly regulate angiogenesis.

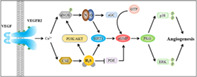

## 1 INTRODUCTION

Angiogenesis refers to the physiological process of forming new blood vessels from existing capillaries or posterior veins of capillaries.^1^, ^2^, ^3^, ^4^ Angiogenesis plays a key role in human health and disease.^5^ Physiological angiogenesis is beneficial to embryonic development, female physiological period, and wound healing. Pathological angiogenesis leads to the occurrence of a variety of diseases. Excessive angiogenesis can lead to cancer, arthritis, psoriasis and blindness, obesity, asthma, atherosclerosis, and some infectious diseases.^6^, ^7^ Insufficient growth or degeneration of blood vessels can cause myocardial hypoxia, cerebral hypoxia, stroke, hypertension, and osteoporosis.^2^, ^8^ These diseases associated with abnormal angiogenesis are collectively referred to as “angiogenesis‐dependent diseases”.^7^ In light of angiogenesis is involved in many physiological and pathological processes, more efforts should be paid to illuminate its occurrence, regulation, and potential therapeutic targets.

Hydrogen sulphide (H_2_S) is a toxic gas with an odour of rotten eggs.^9^, ^10^ It is the third endogenous gas signalling molecule after carbon monoxide and nitric oxide (NO).^11^ H_2_S plays an important role in a variety of physiological and pathological processes.^12^, ^13^, ^14^ Given many studies have focused on H_2_S and angiogenesis, the underlying mechanisms are needed to be further investigated. In this article, we highlight the mechanisms of action of H_2_S in physiological and pathological angiogenesis.

## 2 PRODUCTION AND METABOLISM OF H_2_S

**FIGURE 1:**
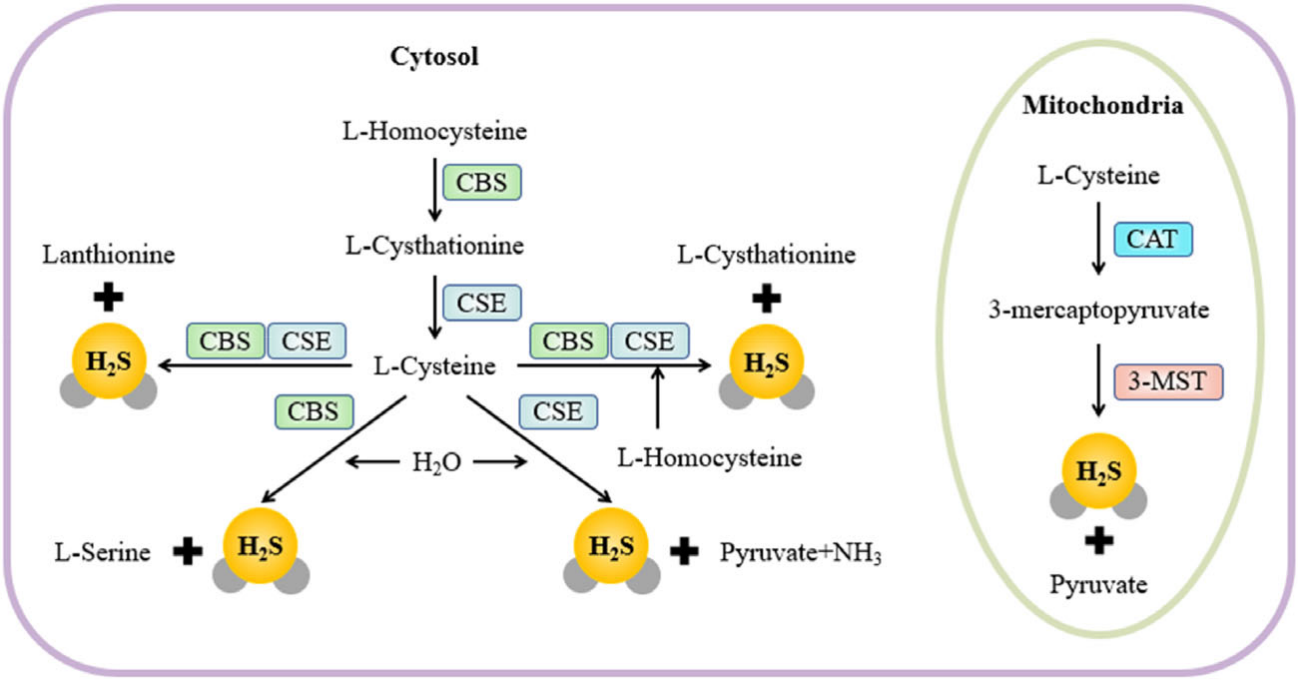
The synthesis of intracellular H_2_S. CBS, CSE, 3‐MST/CAT are enzymes that catalyse the production of H_2_S in cells. CAT, cysteine aminotransferase; 3‐MST, 3‐mercaptopyruvate sulphurtransferase; CSE, cystathionine γ‐lyase; CBS, cystathionine β‐synthase.

In mammals, H_2_S is mainly produced by cystathionine γ‐lyase (CSE), cystathionine β‐synthase (CBS), 3‐mercaptopyruvate sulphurtransferase (3‐MST), and cysteine aminotransferase (CAT)^15^, ^16^, ^17^, ^18^, ^19^ (Figure 1). CSE, CBS, and 3‐MST have different tissue distribution and subcellular localization.^12^ CBS is mainly located in the liver and the central nervous system.^20^ CSE, a predominant source of H_2_S in the cardiovascular system, is localized to cardiomyocytes, vascular endothelial cells, vascular smooth muscle cells, and brown perivascular adipose tissue.^21^, ^22^, ^23^, ^24^, ^25^, ^26^ CSE is also expressed in the peripheral vascular system, such as aorta, pulmonary artery, portal vein, and mesenteric artery.^27^ CBS and CSE are both pyridoxal‐5'‐phosphate (PLP)‐dependent enzymes that produce H_2_S using homocysteine (Hcy) and l‐cysteine as substrates. As a non‐PLP‐dependent enzyme, 3‐MST is mostly located in mitochondria and produces H_2_S with CAT using 3‐mercaptopyruvate (3‐MP) as substrate.^28^ In addition, the three enzymes can be found in the vascular endothelium.^18^, ^29^, ^30^ The lipophilic characteristics of H_2_S and the localization advantage of H_2_S‐producing enzymes together determine that H_2_S plays a vital role in regulating vasodilation, angiogenesis and anti‐endothelial cell senescence.^31^, ^32^, ^33^, ^34^, ^35^, ^36^, ^37^, ^38^, ^39^

Scavenging mechanism contributes to alleviate toxic effects of H_2_S. H_2_S can be oxidized in mitochondria by the sulphide quinone oxidoreductase system to form thiosulphates and sulphates, or methylated in the cytoplasm by thiol‐S‐methyltransferase to form dimethyl sulphide and methanethiol.^40^, ^41^ It can also be bound by methaemoglobin to produce Sul haemoglobin, which is excreted by spleen.^42^

## 3 THE ROLE OF H_2_S IN TUMOUR ANGIOGENESIS

In normal tissues, angiogenesis is regulated by anti‐angiogenic factors and pro‐angiogenic factors to achieve a vascular resting state. A large amount of oxygen and nutrients are needed for tumour division and proliferation. When the tumour exceeds a certain volume, new blood vessels are formed to maintain it. Tumour angiogenesis is the result of increased pro‐angiogenic factors or decreased anti‐angiogenic factors and is a necessary condition for the growth and metastasis of tumour.^43^, ^44^

### 3.1 Hypoxia, hypoxia‐inducible factor 1, and H_2_S

In solid tumours, hypoxia is caused by high oxygen consumption, nutritional deficiency, and accumulation of metabolites. Hypoxia is a typical feature of the tumour microenvironment and is an important reason for malignant transformation of tumours.^45^ Hypoxia‐inducible factor 1 (HIF‐1) is the major factor regulating oxygen homeostasis, which exists in mammalian cells cultured under reduced O_2_ tension. HIF‐1 has a basic‐loop‐helix‐Per‐ARNT‐Sim (PAS) heterodimer structure, consisting of two subunits, HIF‐1α and HIF‐1β.^46^

Under normal O_2_ tension, HIF‐1α and HIF‐2α are hydroxylated by prolyl hydroxylases (PHDs), then bound by the von Hippel Lindau (VHL), and eventually degraded by the ubiquitin‐proteasome system.^47^, ^48^, ^49^ In the hypoxic environment of the tumour, HIF‐1α/HIF‐2α cannot be hydroxylated due to inactivation of PHDs.^50^ Decreased binding of HIFα to VHL promotes the entry of HIF‐1α‐HIF‐1β dimer into the nucleus. The proliferation and migration of tumour cells and tube formation can be promoted by activating the expression of vascular endothelial growth factor (VEGF) and other angiogenesis‐related genes.^51^, ^52^ Therefore, the activation of HIF‐1 is one of the key adaptive response mechanisms of tumours to cope with the hypoxic environment.^53^

In addition to the regulation of the degradation of HIF‐1α by hydroxylases, the protein translation of HIF‐1α in hypoxia is worthy of attention. This process is mediated by the phosphorylation of eukaryotic translation initiation factor 4E binding protein 1 via RAS/RAF/MEK/ERK kinase cascade and the PI3K‐AKT‐mTOR pathway.^52^, ^54^, ^55^

The level of H_2_S is increased under anoxic conditions.^45^, ^56^, ^57^ On the one hand, CSE is promoted by hypoxia to transfer to the mitochondria, where the amount of cysteine is about three times than that in cytoplasmic matrix. Subsequently, the expression of H_2_S in mammals is upregulated via the metabolism of cysteine by CSE.^58^, ^59^ In the mitochondrial matrix, the oxygenation state of the haeme group contained in CBS is the decisive factor for Lon protease to recognize and degrade CBS protein. However, in hypoxia, the deoxygenated haeme group in CBS cannot be recognized by Lon protease, resulting in the accumulation of CBS in mitochondria.^60^ HIF‐1 can also increase the expression of CBS in the cerebellum and the cerebral cortex.^61^ On the other hand, the oxidative metabolism of H_2_S in mitochondria is inhibited.^62^, ^63^ It has been reported that pro‐angiogenic effects of H_2_S are mediated by inhibiting mitochondrial electron transport and oxidative phosphorylation, which increases glycolysis and the production of adenosine triphosphate (ATP).^64^ Therefore, H_2_S can play a cytoprotective role as an oxygen sensor in hypoxia.

Under anoxic conditions, H_2_S has a regulatory effect on HIF‐1. It has been shown that the protein level and activity of HIF‐1 can be increased by endogenous H_2_S and hypoxia in *Caenorhabditis elegans*.^65^ The EGg laying defective (EGL)‐9 is responsible for the hydroxylation of HIF‐1. A negative regulator of EGL‐9, CYSL‐1, is homologous to CBS, can promote H_2_S‐induced HIF‐1 accumulation after hypoxia by interacting with the C‐terminus of EGL‐9.^66^ The expression and stability of HIF‐1α can be enhanced by supplementing H_2_S with diallyl disulphide (DADS).^67^ Under anoxic conditions, H_2_S can also stimulate the expression and activation of HIF‐1 in a NO‐dependent manner.^68^ Similarly, the mRNA and protein levels of HIF‐1 and VEGF are increased by treating brain capillary endothelial cells with sodium hydrosulphide (NaHS), and the binding activity of HIF‐1α is enhanced under anoxic conditions to promote angiogenesis.^69^ The expression of HIF‐1α induced by NaHS is dependent on nuclear factor‐E2‐related factor 2.^70^ However, the translation of HIF‐1α and the expression of HIF‐1 can also be inhibited by H_2_S under hypoxia by enhancing the phosphorylation of eIF2α.^71^ In some cases, HIF‐1 can be inhibited by many H_2_S donors.^72^ In conclusion, the regulation of HIF‐1 by H_2_S plays an important role in angiogenesis.

### 3.2 The pro‐angiogenic effect of H_2_S in cancer

More and more studies have shown that a variety of H_2_S‐producing enzymes are dysregulated in various cancers, and the role of H_2_S in cancer has been extensively studied.^73^, ^74^

Mechanistically, under hypoxic conditions, H_2_S induces the proliferation and migration of endothelial cells (ECs) and promotes tumour angiogenesis by increasing the expression of HIF‐1α and VEGF.^69^ It has been shown that H_2_S can enhance the activity and translation of HIF‐1α. In non‐small cell lung cancer, HIF‐1α is activated by H_2_S via the PI3K‐AKT pathway to regulate the epithelial‐mesenchymal transition and angiogenesis.^75^ H_2_S promotes angiogenesis by downregulating miR‐640 expression and increasing HIF‐1α levels via the VEGFR2‐mTOR pathway.^76^ In addition to increasing the source of HIF‐1α, inhibiting its degradation can also promote angiogenesis. For example, the degradation of HIF‐1α is blocked by pseudo hypoxia because of the lack of VHL in clear cell renal cell carcinoma, the level of H_2_S is increased, which in turn will promote angiogenesis.^77^ H_2_S produced by CBS stimulates angiogenesis by activating AP‐1 to upregulate VEGF in colon cancer.^78^, ^79^ CSE can also promote angiogenesis through the VEGF signalling pathway, which is the key to the metastasis of breast cancer.^80^ 3‐MST has a promoting effect on the migration of vascular ECs cultured in a hypoxic environment.^81^ The intracellular calcium signal is induced by VEGF, resulting in the increase of intracellular Ca^2+^ concentration and promotion of ECs proliferation and migration. Therefore, H_2_S plays an important role in promoting angiogenesis in cancer cells, which can be inhibited by dl‐propargylglycine (PAG), a CSE inhibitor.^82^, ^83^, ^84^

**FIGURE 2:**
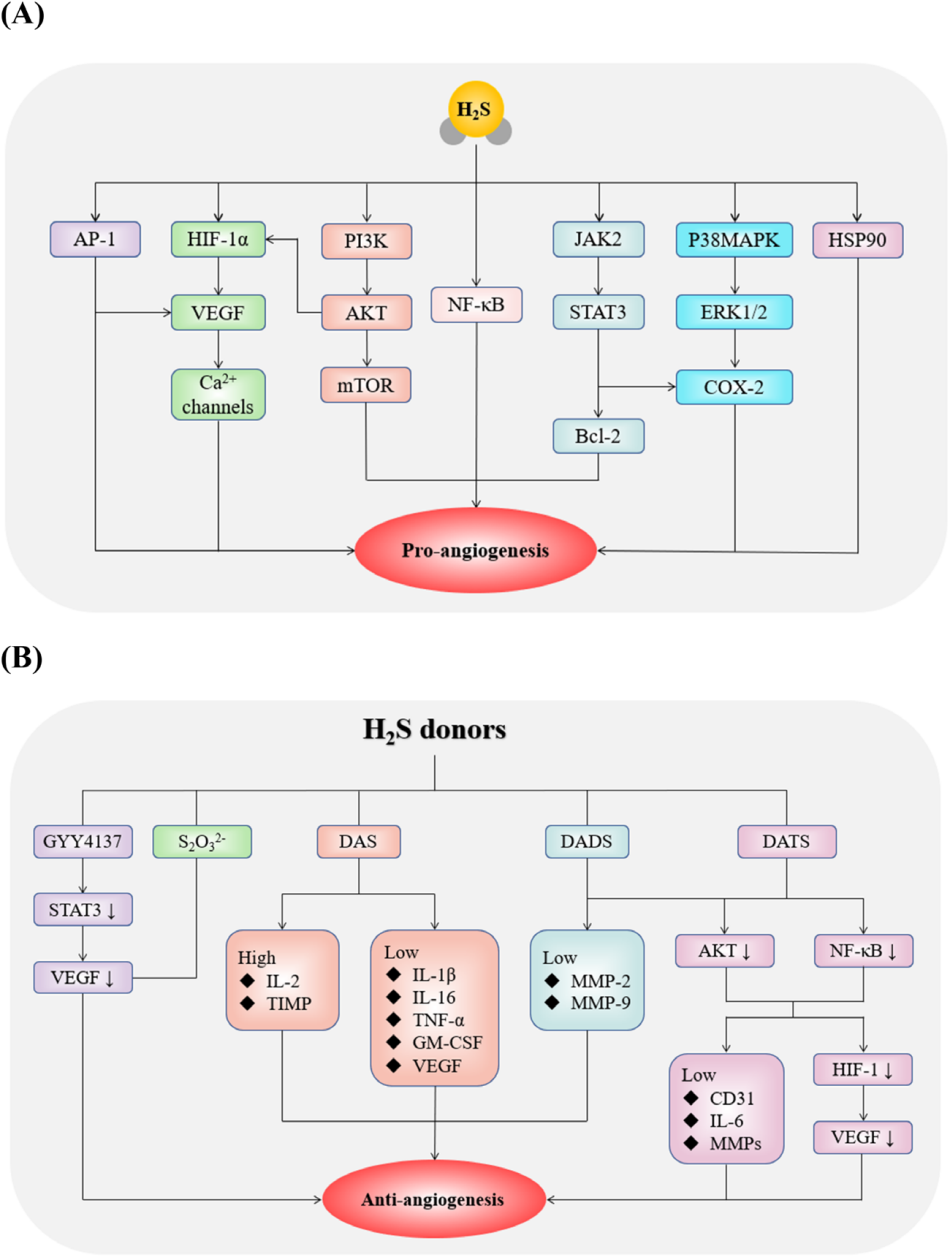
The pro‐angiogenic and anti‐angiogenic roles of H_2_S in cancer progression. (A) Tumour angiogenesis is promoted by H_2_S via stimulating AP‐1, HIF‐1α, PI3K, NF‐κB, JAK2, MAPK and HSP90. (B) H_2_S donors exert anti‐angiogenic effect on the development of cancer. AKT, protein kinase B; AP‐1, activating protein‐1; Bcl‐2, B‐cell lymphoma 2; CD31, platelet/endothelial cell adhesion molecule‐1; COX‐2: cyclooxygenase‐2; DADS, diallyl disulphide; DAS, diallyl sulphide; DATS, diallyl trisulphide; ERK, extracellular signal‐related kinases; GM‐CSF, granulocyte macrophage colony‐stimulating factor; HIF‐1α, hypoxia‐inducible factor‐1α; HSP90, heat‐shock protein 90; IL‐1β, interleukin‐1β; IL‐2, interleukin‐2; IL‐6, interleukin‐6; IL‐16, interleukin‐16; JAK‐2: janus kinase 2; MAPK, mitogen‐activated protein kinase; MMP‐2, matrix metalloproteinase‐2; MMP‐9, matrix metalloproteinase‐9; mTOR, mammalian target of rapamycin; NF‐кB, nuclear factor‐kappa B; PI3K, phosphoinositide 3‐kinase; S_2_O_3_^2−^, thiosulfate; STAT3, signal transducer and activator of transcription 3; TIMP, tissue inhibitor of metalloproteinase; TNF‐α, tumour necrosis factor‐α; VEGF, vascular endothelial growth factor; VEGF, vascular endothelial growth factor.

H_2_S‐induced tumour angiogenesis also involves many other signalling pathways. It has been demonstrated that H_2_S can promote angiogenesis in hepatocellular carcinoma (HCC), glioma, and oesophageal cancer by activating the nuclear factor‐kappa B (NF‐κB), p38 mitogen‐activated protein kinase (MAPK)/ERK1/2‐COX‐2 and HSP90 pathways, respectively.^85^, ^86^, ^87^ The similar effects have been observed in liver cancer and oesophageal cancer via the signal transducer and activator of transcription 3 (STAT3)‐COX‐2 and JAK2/STAT3 pathway^88^, ^89^ (Figure 2).

Given the pro‐angiogenic effect of H_2_S, tumours can be treated by antagonizing this effect. In the presence of non‐organ‐specific cancer prevention molecule, Korean red ginseng (KRGE), the expression levels of CSE and CBS in human umbilical cord blood endothelial cells (HUVECs) are effectively decreased. In addition, the expressions of HIF‐1α and VEGF are significantly reduced, indicating that the antagonism of H_2_S‐induced angiogenesis is the potential mechanism for KRGE to prevent gastric cancer.^90^ The combination of traditional Chinese medicine kelp and curcuma zedoary can inhibit the production of endogenous H_2_S, and the proliferation and metastasis of liver cancer cells are attenuated by downregulating the expression levels of the p‐STAT3/BCL‐2 and VEGF pathways and their downstream key genes p‐ERK1/2 and p‐AKT, indicating that H_2_S plays a key role in the treatment of liver cancer.^91^ In conclusion, H_2_S can act as a promising target of anti‐angiogenetic strategy in cancer treatment.

### 3.3 The anti‐angiogenic effect of H_2_S in cancer

However, it should be noted that angiogenesis can also be inhibited by GYY4137, a donor of H_2_S, which can reduce VEGF and HIF‐1α by blocking the STAT3 pathway in human HCC cells.^92^ In addition, the sustained‐release H_2_S donor, thiosulphate, can reduce the expression of VEGF‐induced CSE and attenuate the proliferation of HUVECs, which plays a therapeutic role in anti‐angiogenesis.^93^

Garlic extracts, such as DADS, diallyl sulphide (DAS), and diallyl trisulphide (DATS), are widely used as H_2_S donors, and their anti‐angiogenic effects have been applied to cancer treatment.^93^ In Ehrlich ascites tumour‐bearing mice, DAS can inhibit angiogenesis in a dose‐dependent manner.^94^, ^95^ DADS acts as an inhibitor of angiogenesis by inhibiting the activation of matrix metalloproteinases during endothelial morphogenesis.^96^ The novel pro‐angiogenic effect of H_2_S is dependent on AKT phosphorylation.^97^ DATS has been extensively studied as an anti‐cancer and chemopreventive agent.^98^, ^99^, ^100^ Angiogenesis could be inhibited by DATS via inactivating AKT and downregulating VEGF and VEGFR2 in HUVECs, reducing the activation of AKT and NF‐κB in prostate cancer, inactivating the Wnt/β‐catenin signal transduction in glioma, and reducing the synthesis of HIF‐1α in breast cancer^72^, ^101^, ^102^, ^103^, ^104^ (Figure 2).

In conclusion, H_2_S has the dual effects of pro‐angiogenesis and anti‐angiogenesis. We speculate that the effect of H_2_S on tumour angiogenesis follows a bell‐shaped dose response, which may be regulated by the concentration of H_2_S. At low concentrations, H_2_S exhibits a protective role in promoting angiogenesis, while at high concentrations, it is opposite. It has been shown that NaHS acts as a double‐edged sword in human HCC cells through the PTEN/AKT and the EGFR/ERK/MMP‐2 signalling pathway. Angiogenesis is promoted by 25–100 μM NaHS, while 800–1000 μM NaHS shows opposite effect.^105^

## 4 THE ROLE OF H_2_S IN ANGIOGENESIS IN CARDIOVASCULAR DISEASES

Numerous studies have shown that H_2_S is an effective cardiovascular protective agent, which plays a role in promoting cardiovascular homeostasis and health through vasodilation, angiogenesis, inflammation, oxidative stress, and apoptosis.^27^, ^41^, ^106^, ^107^ As an endogenous gas stimulator of angiogenesis, H_2_S has the effect of promoting angiogenesis in cardiovascular diseases such as ischaemic diseases, myocardial infarction, heart failure and atherosclerosis.^29^, ^108^

### 4.1 Interaction between H_2_S and VEGF

The interaction between H_2_S and VEGF can promote angiogenesis. CSE expression is enhanced in a calcium‐dependent manner via VEGF‐VEGFR2 binding, thus increasing H_2_S levels.^109^ In turn, VEGFR2 is activated by H_2_S and the VEGF‐VEGFR2 binding is enhanced by breaking the Cys1045‐Cys1024 disulphide bond of VEGFR2.^110^

As a therapeutic target, angiogenesis plays an irreplaceable role in the recovery of ischaemic diseases.^6^, ^111^ NaHS can promote the growth of collateral vessels, increase regional tissue blood flow in a rat with unilateral hindlimb ischaemia. These effects may be mediated by the interaction of upregulated VEGF in skeletal muscle cells, VEGFR2 in vascular endothelial cells, as well as the downstream signal transduction element AKT.^37^, ^112^ S‐propargyl‐cysteine (SPRC), a novel water‐soluble regulator, can enhance the interaction between VEGFR2 and growth factor receptor‐bound protein 2 by activating CSE to produce endogenous H_2_S. The phosphorylation level of STAT3 is induced. STAT3 moves from the cytoplasmic matrix to the nucleus via the JAK‐STAT3 pathway, activates VEGF promoter, and promotes cell proliferation, migration and tube formation. Pathologically, after ligation of the coronary artery or the left femoral artery, treatment with SPRC can promote angiogenesis and alleviate ischaemia.^113^ In cerebral ischaemic diseases, cerebral ischaemia can be improved by H_2_S via promoting the phosphorylation of AKT and ERK, as well as increasing the expression of VEGF and angiopoietin‐1 (Ang‐1).^114^ Therefore, H_2_S can promote angiogenesis in ischaemic diseases via the AKT or JAK2‐STAT3 pathway, which is mediated by VEGFR2.

### 4.2 Interaction between H_2_S and NO

**FIGURE 3:**
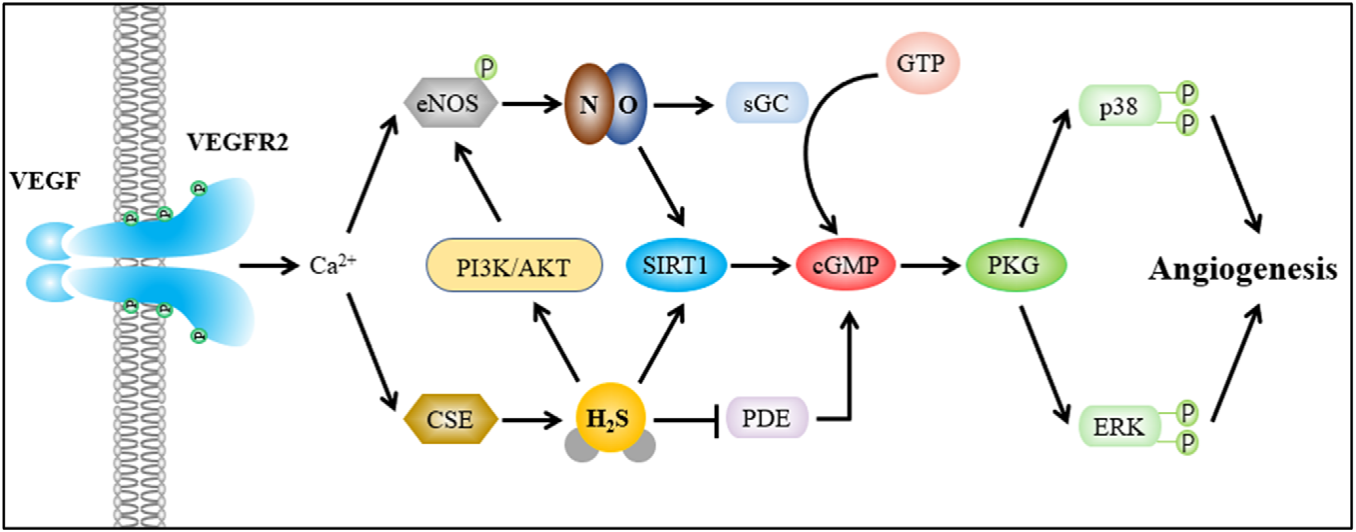
H_2_S and NO interact and depend on each other to jointly regulate angiogenesis. AKT, protein kinase B; cGMP, cyclic guanosine monophosphate; CSE, cystathionine γ‐lyase; eNOS, endothelial nitric oxide synthase; ERK, extracellular signal‐related kinases; GTP, guanosine triphosphate; sGC, soluble guanylate cyclase; SIRT1, sirtuin 1; PDE, phosphodiesterase; PI3K, phosphoinositide 3‐kinase; PKG, protein kinase G; VEGF, vascular endothelial growth factor.

In the cardiovascular and cerebrovascular system, H_2_S and NO can interact and depend on each other to regulate angiogenesis.^115^, ^116^, ^117^, ^118^, ^119^, ^120^ During tissue ischaemia, H_2_S can increase the phosphorylation level of endothelial nitric oxide synthase (eNOS) at its activating site S1177 via the PI3K/AKT‐dependent pathway.^115^, ^116^ ATP‐sensitive potassium channels mediate partial vascular functions of H_2_S and participate in the activation of AKT.^121^, ^122^ The production of NO is increased in ECs and vascular tissues via eNOS or xanthine oxidase‐mediated nitrite reduction mechanism.^68^ Soluble guanylate cyclase is bonded and activated by NO, which can catalyse guanosine triphosphate into cyclic guanosine monophosphate (cGMP).^123^ H_2_S can also increase cGMP by inhibiting the activity of phosphodiesterase.^124^ The cGMP‐dependent protein kinase G (PKG) is activated, which could contribute to the proliferation and migration of ECs or the expression of growth factors and angiogenesis through the downstream MAPK pathway.^125^, ^126^, ^127^, ^128^ In addition to the cGMP/PKG/MAPK pathway, NO also modulates HIF‐1α and VEGF‐dependent angiogenesis, thus stimulating ischaemic vascular remodelling^68^, ^129^ (Figure 3).

### 4.3 H_2_S in ischaemic diseases

H_2_S has shown the protective role in limb ischaemic diseases by regulating angiogenesis. Under physiological conditions, a novel H_2_S–NO hybrid molecule, ZYZ‐803, could stimulate the expression of CSE and enhance the activity of endothelial eNOS. NO and H_2_S are slowly released and mediate the increase of angiogenesis in rat aortic rings and mouse ischaemic hindlimb models via the SIRT1/VEGF/cGMP pathway.^130^, ^131^ ZYZ‐803 can also promote angiogenesis in mice with femoral artery ligation via the STAT3/Ca^2+^/CaM‐dependent protein kinase II pathway.^132^ Similar to ZYZ‐803, H_2_S prodrug SG‐1002 can promote angiogenesis in a porcine model of acute limb ischaemia and improve peripheral arterial disease by increasing the signal transduction of H_2_S and NO in the circulation.^133^ DATS can enhance ischaemia‐induced angiogenesis and stimulate the phosphorylation of AKT and eNOS through the AKT‐eNOS signalling pathway in mice with unilateral hindlimb ischaemia (HLI).^134^ Another study has shown that the intramuscular injection of a poly (d,l‐lactic‐co‐glycolic acid) microparticle system that contains DATS can promote therapeutic angiogenesis and prevent apoptosis and tissue necrosis in a mouse model of limb ischaemia.^135^ Taken together, in limb ischaemic diseases, H_2_S can mediate angiogenesis by activating the eNOS/NO pathway.

### 4.4 H_2_S in heart diseases

H_2_S also has protective effect on myocardial ischaemia, myocardial infarction, and heart failure via angiogenesis.^136^, ^137^ Myocardial ischaemia is usually caused by insufficient blood supply to the myocardium because of coronary stenosis. GYY4137, a slow‐releasing H_2_S donor, can attenuate adverse remodelling and promote angiogenesis after ischaemia.^138^

VEGFR1 and VEGFR2, two receptor tyrosine kinases, can bind VEGF with high affinity, stimulate ECs proliferation, and enhance angiogenesis. Treatment with H_2_S improves the cardiac function by increasing the expressions of VEGF, VEGFR1, and VEGFR2 but decreasing the levels of anti‐angiogenic factors such as angiostatin and endostatin.^139^, ^140^, ^141^, ^142^, ^143^ The downregulation of CBS, CSE, and 5‐methylenetetrahydrofolate reductase caused by myocardial infarction will result in hyperhomocysteinemia (HHcy) and inhibit ECs proliferation, migration and angiogenesis.^144^, ^145^, ^146^ HHcy can also inhibit angiogenesis by antagonizing the angiogenic signalling pathway of PPAR‐γ/VEGF axis, which can be improved by GYY4137 via enhancing PPAR‐γ‐VEGF‐eNOS‐NO signal transduction in skeletal muscle cells.^147^ It has been shown that GYY4137 can also promote cerebral angiogenesis in zebrafish through the eNOS/NO pathway.^148^ In addition, HHcy is an independent risk factor for atherosclerosis with the typical feature of endothelial dysfunction. CSE is expressed in the microvessels of human atherosclerotic plaques and participates in micro‐angiogenesis, which can improve endothelial function.^149^, ^150^, ^151^ In the renal vasculature, the disturbance of Hcy metabolism will lead to renovascular hypertension, which can be improved by the conversion of Hcy to H_2_S via the AKT/FoxO3 pathway after gene therapy with CBS, CSE, and 3‐MST.^152^, ^153^

Heart failure may be caused by myocardial infarction by upregulation of the levels of MMP‐9 and anti‐angiogenic factors.^154^ Treatment with H_2_S donors could inhibit the transition from compensatory hypertrophy to heart failure by inducing the production of MMP‐2, inhibiting the expression of TIMP‐3 and MMP‐9, and promoting the synthesis of VEGF.^155^ H_2_S can attenuate cardiac dysfunction after heart failure via the VEGF‐eNOS‐NO pathway to promote angiogenesis and the GPx‐1‐HO‐1 pathway to counteract oxidative stress.^156^ HSD‐R, a novel H_2_S donor, can achieve myocardial protection by inhibiting local inflammation, reducing cardiomyocyte apoptosis, and promoting angiogenesis, indicating that HSD‐R can act as a promising therapeutic agent for myocardial infarction and other ischaemic diseases.^157^

In conclusion, in addition to its pro‐angiogenic effect in myocardial infarction, H_2_S can treat atherosclerosis and renovascular hypertension by improving HHcy‐induced angiogenic disorders. H_2_S also provides a clinical possibility for the prevention and treatment of heart failure by upregulating MMP2, downregulating MMP‐9 and TIMP‐3, and promoting angiogenesis through the eNOS‐NO pathway.

## 5 THE ROLE OF H_2_S IN DIABETIC ANGIOGENESIS

Diabetes is a metabolic disease characterized by elevated blood glucose levels and is a main risk factor for vascular diseases. The vascular complications, mainly led by abnormal angiogenesis, can be divided into two types: macrovascular complications (coronary and peripheral arterial diseases, cerebrovascular) and microvascular complications (nephropathy, neuropathy, and retinopathy).^158^

Refractory wound lesions are easily induced by abnormal angiogenesis in diabetic patients, and the basis of wound healing depends on angiogenesis.^159^, ^160^, ^161^, ^162^, ^163^ Impaired angiogenesis induced by diabetes is related to Ang‐1/Tie 2 signalling.^164^ The decreased expressions of many vascular growth factors such as platelet‐derived growth factor and VEGF are involved in the impaired angiogenesis caused by hyperglycaemia/diabetes.^165^ H_2_S promotes angiogenesis in diabetic db/db mice by increasing the expression levels of VEGF and Ang‐1 in wound skin tissues and endothelial progenitor cells (EPCs).^159^, ^166^ The similar effect has been observed by NaHS treatment in ob/ob mice.^167^ Wound healing can be improved by NaHS in streptozotocin‐induced diabetic rats, which is associated with enhanced angiogenesis and increased levels of intercellular adhesion molecule‐1 (ICAM‐1) and VEGF.^168^ The occurrence of diabetic vascular complications is related to endothelial dysfunction, and angiogenesis is reduced by impaired function of ECs.^169^, ^170^ In Type 2 diabetes, H_2_S improves wound healing by restoring the function of EPCs and activating Ang‐1.^159^ H_2_S plays a key role in maintaining the function of ECs and angiogenesis in diabetes.^171^ EC‐related angiogenic property is stimulated by H_2_S via the K_ATP_ channel/MAPK pathway.^29^ H_2_S can protect HUVECs against high glucose‐induced injury. On the one hand, H_2_S upregulates the miR‐126‐3p level and recovers ECs migration via downregulating the DNMT1 protein level induced by high glucose, thereby improving the impaired angiogenesis induced by high glucose.^172^, ^173^ On the other hand, the activation of PI3K/AKT/eNOS pathway is essential for H_2_S to prevent HUVECs from injury.^174^ Accordingly, H_2_S‐releasing micelles are prepared to enhance HUVECs migration and tube formation by delivering H_2_S.^175^ The downregulation of CSE/H_2_S system is related to diabetes‐impaired angiogenesis. The activation of local CSE‐H_2_S‐VEGF axis may contribute to pro‐angiogenic effects of DH injection in diabetic hind limb ischaemia model mice.^176^ Overexpression of CSE or treatment with DATS has therapeutic actions on diabetic mouse models by promoting revascularization in ischaemic tissue via eNOS/NO signalling pathway.^177^, ^178^ Furthermore, impaired angiogenesis in hyperglycaemia/diabetes is associated with impaired pro‐angiogenic properties of 3‐MP. A 3‐MST stimulator/cofactor, lipoic acid, can restore or improve the ability of 3‐MP to stimulate angiogenesis and wound healing in hyperglycaemia.^179^ Therefore, 3‐MST pathway has the potential to promote angiogenesis.^180^ These findings suggest that H_2_S can improve angiogenesis in diabetes.

H_2_S donors have shown angiogenic response in diabetic models. Islet transplantation into subcutaneous polymer scaffolds has been proved to be able to induce normoglycaemia in type 1 diabetes models. The fast‐releasing H_2_S donor, NaHS (25 or 50 μmol/kg), is intraperitoneally injected into the nude mice implanted with subcutaneous scaffolds. After 63 days, the mRNA expression of angiogenesis marker CD105 and the number of CD31 positive vessels are significantly higher than those in the control group, indicating that H_2_S can promote vascularization of subcutaneous scaffolds.^181^ However, the release of H_2_S by NaHS is fast and difficult to control, which is prone to cause cytotoxicity due to high local concentration. To solve this problem, a microparticle system (NaHS@MPs) encapsulated NaHS has been designed. The system can continuously provide H_2_S and promote the proliferation, migration of epidermis cells/ECs and angiogenesis by extending the activation of p38 and ERK1/2, thereby accelerating the wound healing in diabetic mice.^182^

In summary, H_2_S can upregulate VEGF, Ang‐1, and ICAM‐1, as well as accelerate cell proliferation, migration and tube formation by activating the K_ATP_ channel/MAPK pathway and the PI3K/AKT/eNOS pathway, thereby improving wound healing and treating diabetic vascular complications.

## 6 THE ROLE OF H_2_S IN ANGIOGENESIS OF THE REPRODUCTIVE SYSTEM

Angiogenesis is a normal physiological phenomenon of endometrial regeneration during menstrual cycles and pregnancy, which is related to the elevated level of H_2_S.^183^ CSE and CBS are expressed in human intrauterine tissues.^184^ During the menstrual cycle and pregnancy, the endometrial CBS‐H_2_S production is stimulated due to the increase of oestrogen level, which promotes human endometrial angiogenesis.^185^ Endogenous H_2_S is required for a healthy placental vasculature. Pregnancy could increase the level of H_2_S in human uterine artery endothelial cells and stimulate the production of placental VEGF in placental trophoblasts, which is mediated by the MAPK3/1 and AKT1‐NOS3 pathways.^186^, ^187^, ^188^ In maternal obesity, DATS has been used to increase the level of H_2_S in serum and placenta of mice, which can promote placental angiogenesis by regulating lipid metabolism, reducing inflammation, and activating the PI3K/AKT pathway.^189^

Abnormal placental angiogenesis is associated with pre‐eclampsia.^190^ Pre‐eclampsia is a pregnancy‐related vascular disease and is the leading cause of maternal and foetal morbidity and mortality. Before the onset of pre‐eclampsia, the circulating levels of soluble endoglin (sEng) and soluble FMS‐like tyrosine kinase‐1 (sFlt‐1) are increased, and then VEGF and placental growth factor are decreased, thus leading to pre‐eclampsia.^191^, ^192^, ^193^, ^194^ The plasma H_2_S level in women with pre‐eclampsia is reduced. After treatment with GYY4137, sFlt‐1 and sEng are inhibited and the effect of PAG on foetal growth can be weakened.^195^, ^196^ Taken together, the decrease of CSE activity is associated with the pathogenesis of pre‐eclampsia.

In contrast to the female reproductive system, although H_2_S plays an important role in various male sex organs such as the penis, prostate, and vas deferens, the role of H_2_S in the angiogenesis in male reproductive system is still unclear and needs further exploration to provide therapeutic targets for the related diseases.^197^

## 7 CONCLUSIONS, LIMITATIONS, AND FUTURE DIRECTIONS

Numerous studies have shown that H_2_S plays an important role in the angiogenesis of ECs. In this review, we summarize the roles of H_2_S in physiological and pathological angiogenesis (Table 1). In tumours, H_2_S has a pro‐angiogenic effect by activating HIF‐1 via the RAS/RAF/MEK/ERK cascade and the PI3K‐AKT‐mTOR pathway. VEGF is a crucial factor in H_2_S‐induced angiogenesis. In cardiovascular system, in addition to the AKT and JAK‐STAT3 pathway mediated by VEGFR2, H_2_S can stimulate angiogenic activity by interacting with NO. H_2_S has achieved good therapeutic effects in diabetic vascular complications by activating K_ATP_ channel/MAPK or PI3K/AKT/eNOS pathway to upregulate pro‐angiogenic factors. Although H_2_S can inhibit angiogenesis by blocking the STAT3 pathway or inactivating the Wnt/β‐catenin cascade in tumours, the underlying mechanism needs to be further investigated.

**TABLE 1:** The pro‐angiogenic and anti‐angiogenic roles of H_2_S in various diseases.

Diseases		Mechanisms	Effects	References
Cancer	Non‐small cell lung cancer	Activation of HIF‐1α by the PI3K‐AKT pathway	Pro‐angiogenesis	75
	Clear cell renal cell carcinoma	Blocking the degradation of HIF‐1, elevating the level of H_2_S		77
	Colon cancer	Up‐regulating VEGF by activating AP‐1		78, 79
	Breast cancer	Through the VEGF signalling pathway		80
	Hepatocellular carcinoma	Activating NF‐κB and STAT3‐COX‐2 signalling pathways		85, 88
	Glioma	Activating the p38 MAPK/ERK1/2‐COX‐2 pathway		86
	Oesophageal cancer	Activating HSP90 pathway and JAK2/STAT3 signalling pathway		87, 89
Cardiovascular diseases	Limb ischaemia	Activating VEGF via JAK‐STAT3 pathway; promoting angiogenesis through the SIRT1/VEGF/cGMP pathway, the STAT3/Ca^2+^/CaMKII pathway or the AKT‐eNOS signalling pathway		113, 130, 131, 132, 133, 134
	Cerebral ischaemia	Increasing expression of VEGF and Ang‐1 by promoting phosphorylation of AKT and ERK		114
	Myocardial ischaemia	Attenuating adverse remodelling		138
	Myocardial infarction	Increasing pro‐angiogenic growth factors and decreasing anti‐angiogenic growth factors		139, 140, 141, 142, 143
	Heart failure	Increasing MMP‐2 and VEGF, inhibiting MMP‐9 and TIMP3; By the VEGF‐eNOS‐NO pro‐angiogenic pathway		155, 156
	HHcy	Enhancing VEGF‐eNOS‐NO signal transduction; converting Hcy to H_2_S via the AKT/FoxO3 pathway		152, 153
Diabetes		Up‐regulating Ang‐1, VEGF, ICAM‐1, miR‐126‐3p; Activating the K_ATP_ channel/MAPK pathway or the PI3K/AKT/eNOS pathway		29, 159, 166, 167, 168, 172, 173, 174
Pre‐eclampsia		Limiting sFlt‐1 and sEng, increasing VEGF and PlGF		195, 196
Cancer	Hepatocellular carcinoma	Blocking the STAT3 pathway and downregulating VEGF	Anti‐angiogenesis	92
	Ehrlich ascites tumour	Up‐regulating of IL‐2 and TIMP, down‐regulating of IL‐1β, IL‐16, TNF‐α, GM‐CSF and VEGF		94, 95
	Prostate cancer	Inhibiting the activation of AKT and NF‐κB		102, 103
	Glioma	Inactivating the Wnt/β‐catenin signal transduction		104
	Breast cancer	Reducing the synthesis of HIF‐1α		72

Abbreviations: AP‐1, activating protein‐1; AKT, protein kinase B; CaMKII, CaM‐dependent protein kinase II; cGMP, cyclic guanosine monophosphate; COX‐2, cyclooxygenase‐2; eNOS, endothelial nitric oxide synthase; ERK, extracellular signal‐regulated kinase; FoxO3, Fork‐head Box O3; GM‐CSF, granulocyte macrophage colony‐stimulating factor; HIF‐1α, hypoxia‐inducible factor‐1α; ICAM‐1, intercellular adhesion molecule‐1; IL‐1β, interleukin‐1β; IL‐2, interleukin‐2; IL‐16, interleukin‐16; JAK2, janus kinase 2; MAPK, mitogen‐activated protein kinase; MMP‐2, matrix metalloproteinase‐2; MMP‐9, matrix metalloproteinase‐9; NF‐κB, nuclear factor‐kappa B; NO, nitric oxide; PI3K, phosphoinositide 3‐kinase; PlGF, placental growth factor; sEng, soluble endocrine hormone; sFlt‐1, soluble FMS‐like tyrosine kinase‐1; SIRT1, sirtuin 1; STAT3, signal transducer and activator of transcription 3; TIMP, tissue inhibitor of metalloproteinase; TNF‐α, tumour necrosis factor‐α; VEGF, vascular endothelial growth factor.

Nowadays, the role of H_2_S in angiogenesis is widely studied in experimental models, which has made notable achievements. For example, H_2_S can promote cell proliferation, migration and tube formation. In addition, whether H_2_S plays a certain role in the degradation of the vascular basement membrane and the activation of ECs need to be explored. It is yet unknown how H_2_S can affect the metabolism of ECs to achieve the physiological balance of blood vessels. Whether H_2_S participates in the quiescence or maturation of vessels also needs to be further investigated. In addition to the concentration of H_2_S, what other factors affect the dual roles of H_2_S in tumour angiogenesis may be a novel direction. The anti‐angiogenic effect of H_2_S is mainly studied using H_2_S donors, and the mechanism is worth exploring. Whether the anti‐angiogenic effect of H_2_S can be reversed by inhibiting H_2_S‐producing enzymes remains unclear. The molecules that can affect angiogenesis by interacting with H_2_S could be further identified.

In conclusion, recent studies have demonstrated the role of H_2_S in angiogenesis. However, the underlying mechanism has been partially demonstrated and needs to be elucidated more systematically and completely. Pharmacological inhibition and silencing endogenous H_2_S synthase have been used in the treatment of excessive angiogenesis‐related diseases. Supplementation of substrates and overexpression of H_2_S‐producing enzymes contribute to wound healing and the improvement of ischaemic diseases. Therefore, the regulation of H_2_S production is a potential therapeutic approach for angiogenesis‐dependent diseases. Novel H_2_S donors and/or inhibitors can be developed in the treatment of angiogenesis‐associated diseases.

## AUTHOR CONTRIBUTIONS

*Conceptualization*: Yuan‐Kun Zhai, Xin‐Ying Ji, and Dong‐Dong Wu. *Data curation*: Yan‐Xia Zhang, Mi‐Rong Jing, Chun‐Bo Cai, Shuai‐Gang Zhu, Chao‐Jing Zhang, and Qi‐Meng Wang. *Funding acquisition*: Xin‐Ying Ji and Dong‐Dong Wu. *Writing‐original draft*: Yan‐Xia Zhang. *Visualization and supervision*: Yuan‐Kun Zhai, Xin‐Ying Ji, and Dong‐Dong Wu. *Writing‐review and editing*: Yan‐Xia Zhang and Dong‐Dong Wu.

## CONFLICT OF INTEREST

The authors declare no potential conflict of interest.

## Data Availability

Data sharing is not applicable to this article as no new data were created or analyzed in this study.
